# The Applications of Machine Learning in the Management of Patients Undergoing Stem Cell Transplantation: Are We Ready?

**DOI:** 10.3390/cancers17030395

**Published:** 2025-01-25

**Authors:** Luca Garuffo, Alessandro Leoni, Roberto Gatta, Simona Bernardi

**Affiliations:** 1 Unit of Blood Disease and Stem Cell Transplantation, Department of Clinical and Experimental Sciences, University of Brescia, ASST Spedali Civili, 25123 Brescia, Italy; luca.garuffo@unibs.it (L.G.); simona.bernardi@unibs.it (S.B.); 2 CREA (Centro di Ricerca Emato-Oncologica AIL), ASST Spedali Civili of Brescia, 25123 Brescia, Italy; 3 Department of Clinical and Experimental Sciences, University of Brescia, 25123 Brescia, Italy; roberto.gatta@unibs.it; 4 National Center for Gene Therapy and Drugs Based on RNA Technology—CN3, 35122 Padua, Italy

**Keywords:** allogeneic stem cell transplantation, artificial intelligence, machine learning, GVHD, SVM, donor selection, risk stratification

## Abstract

Hematopoietic stem cell transplantation (HSCT) is a life-saving therapy for hematologic malignancies, such as leukemia and lymphoma and other severe conditions but is associated with significant risks, including graft versus host disease (GVHD), relapse, and treatment-related mortality. The increasing complexity of clinical, genomic, and biomarker data has spurred interest in machine learning (ML), which has emerged as a transformative tool to enhance decision-making and optimize outcomes in HSCT. This review examines the applications of ML in HSCT, focusing on donor selection, conditioning regimen, and prediction of post-transplant outcomes. Machine learning approaches, including decision trees, random forests, and neural networks, have demonstrated potential in improving donor compatibility algorithms, mortality and relapse prediction, and GVHD risk stratification. Integrating “omics” data with ML models has enabled the identification of novel biomarkers and the development of highly accurate predictive tools, supporting personalized treatment strategies. Despite promising advancements, challenges persist, including data standardization, algorithm interpretability, and ethical considerations regarding patient privacy. While ML holds promise for revolutionizing HSCT management, addressing these barriers through multicenter collaborations and regulatory frameworks remains essential for broader clinical adoption. In addition, the potential of ML can cope with some challenges such as data harmonization, patients’ data protection, and availability of adequate infrastructure. Future research should prioritize larger datasets, multimodal data integration, and robust validation methods to fully realize ML’s transformative potential in HSCT.

## Simple Summary

Hematopoietic stem cell transplantation (HSCT) is a critical treatment for several immunological and hematological conditions, but it carries significant risks, such as graft versus host disease (GvHD). Machine learning (ML) is emerging as a powerful tool to improve decision-making in multiple aspects related to HSCT, including donor selection, conditioning regimens, and outcome prediction. Techniques such as decision trees (DT), random forests (RF), and neural networks (NN) improve donor matching, predict mortality and relapse, and stratify GvHD risk. The integration of genomic and biomarker data with ML has enabled personalized treatment strategies. However, challenges such as data standardization, transparency of algorithms, and ethical concerns need to be addressed. Advancing HSCT with ML requires larger datasets, multimodal integration, and robust validation.

## 1. Introduction

### 1.1. Hematopoietic Stem Cells Transplantation: Background

Hematopoietic stem cell transplantation (HSCT) is a life-saving therapeutic procedure primarily used to treat hematologic malignancies such as leukemia, lymphoma, myelofibrosis, and multiple myeloma (MM), as well as other disorders including severe aplastic anemia and certain inherited immune deficiencies [1,2,3]. Hematopoietic stem cells (HSCs) are multipotent cells capable of differentiating into all blood cell lineages and replenishing the bone marrow after damage from chemotherapy or radiation therapy [4]. These cells are critical for the regeneration of the patient’s immune and hematopoietic systems following the myeloablative treatments that precede the transplant procedure [5].

Depending on the subject from which the stem cells are harvested, it is possible to distinguish two types of HSCT: the autologous HSCT (auto-HSCT), where the patient is infused with his own stem cells, and the allogeneic transplant (allo-HSCT), where the stem cells are harvested from a matched or non-matched donor and then infused in the patient [6].

Despite the efficacy of HSCT in treating these conditions, the procedure is associated with several significant risks and challenges, including graft versus host disease (GVHD), graft failure, relapse of the underlying disease, and other transplant-related complications such as infections, mucositis, et cetera.

### 1.2. Current Challenges in Hematopoietic Stem Cell Transplantation

In the context of allo-HSCT, one of the most pressing challenges is the accurate prediction and mitigation of adverse post-transplant outcomes, one of which, as well as the most frequent and also potentially lethal, is the graft versus host disease (GVHD), a pathology in which the donor cells attack the recipient’s tissues, increasing the risk of morbidity and mortality. Two types of GVHD can be distinguished: acute (aGVHD) and chronic (cGVHD). This distinction was previously based on the onset timing (aGVHD sets in before 100 days and cGVHD sets in after 100 days post-HSCT), but now these pathologies are defined based on their features. aGVHD targets the skin, liver, and gastrointestinal tract, while on the other hand, cGVHD can affect nearly any organ system [7]. In addition, we can also have late-onset acute GVHD and overlap syndrome. The first has the same clinical features as aGVHD but manifests itself 100 days after transplantation, while the second can occur at any time after transplantation with clinical features that are typical of both aGVHD and cGVHD [8]. The selection of a suitable donor, which traditionally focuses on human leukocyte antigen (HLA) compatibility, is another critical factor that directly impacts the likelihood of transplant success. However, even in well-matched donor–recipient pairs, complications can arise due to a variety of genetic, clinical, and environmental factors, and in some cases, a perfect HLA matching does not substantially affect the outcomes [9]. Additionally, the individualized prediction of patient responses to conditioning regimens and immunosuppressive therapies remains a major unmet need in the clinical management of HSCT. In fact, patients need to undergo a conditioning phase in which the bone marrow is prepared to receive the new stem cells. According to the European Bone Marrow Transplantation (EBMT) guidelines, depending on the dose and the type of drug used during the conditioning, the patient can be treated with a low, intermediate, or high intensity regimen, with intensity being its myeloablative power [10,11].

Predictive models based on conventional clinical variables (disease, age, comorbidities, lifestyle, and so on), while helpful, often fall short of accurately forecasting these complex outcomes due to the highly heterogeneous nature of the patient population and the multifactorial risks involved. This has spurred interest in applying advanced computational approaches, such as machine learning, to refine outcome predictions and improve clinical decision-making [12,13].

### 1.3. The Emerging Role of Machine Learning in Medicine

In recent years, machine learning has revolutionized many aspects of personalized medicine by enabling the analysis of large, complex datasets to uncover patterns that may not be evident to clinicians [14]. In the context of HSCT, ML algorithms offer the potential to improve donor selection, predict post-transplant complications, and personalize conditioning regimens, all of which are key to optimizing patient outcomes.

Moreover, the integration of “omics” data (e.g., genomics, proteomics, and metabolomics) with machine learning has opened new avenues for identifying novel biomarkers of transplant success or failure [15]. These approaches hold promise for tailoring treatment strategies to the individual patient [16], minimizing adverse outcomes, and ultimately improving long-term survival rates. Donor selection is an important challenge because even in a totally matched patient–donor pair, the HSCT failure rate is considerable high, highlighting the necessity of a deeper study of the biological features of both the patient and the donor. Multi-omics studies generate a high volume of data, which required specific and complex ML models, so that, also for HSCT, it is possible to move towards a more data-driven and personalized approach to patient care.

## 2. Machine Learning Overview

It is not always easy to draw a clear distinction between conventional statistical models and machine learning. Considering, for example, “Statistics is the art of learning from data” [17] and machine learning as a branch of artificial intelligence (AI), the main difference concerns who is learning (human or machine), and many techniques are shared between them. For example, logistic regression is considered as a machine learning approach in one of the key academic textbooks on AI [18], even if it is clearly and widely used in statistics too. It is not the aim of this article to delve into the distinction between statistics and machine learning. In this work, we will refer to the general concept of machine learning as it is commonly presented in the literature. From this perspective, we can roughly observe that, for example, compared to the traditional statistical approach, it tends to be more data-driven (requiring less prior knowledge), and it is capable of easily training highly non-linear models. However, this comes at the cost of greater difficulty in extracting human-interpretable knowledge from the data (particularly in the case of neural networks) and significant challenges in model validation (mainly due to the difficulty of accurately estimating sampling performance a priori). For example, some linear models, such as Logistic Regression and Lasso, can preserve the *p*-value as a measure of the importance of a variable. Other techniques, such as decision trees or random forests, use other indicators, such as the mean GINI index, which measures the inequality among the values of a frequency distribution. k-Nearest Neighbors (kNN) mostly adopts confusion matrix performance indicators (such as accuracy, F1 score, etc.), while for Artificial Neural Networks (ANNs), there are many options, such as measuring the relative importance of explanatory variables [19].

ML algorithms can be supervised or unsupervised: the former uses labelled data to train models for prediction while the latter uses unsupervised learning works with unlabeled data to discover patterns or structures, such as clusters or associations, without predefined outputs.

Among the algorithms commonly used for machine learning, for the purposes of this work, we can distinguish between the categories outlined in Table 1.

Another remarkable point concerns validation. Because ML does not have a preliminary estimation of the sample size (with the exception of some empirical approaches that are debated in the literature), the final model validation is pivotal. In the literature there are many approaches, for example validation on the internal testing set (the weakest) or the two separated sets, for training and testing, respectively. Other approaches are possible: with cross-fold validation, the dataset is split into multiple subsets or folds. Here, the model is trained on some folds and tested on the remaining folds, rotating this process to ensure each fold is used for both training and testing. Another approach is the Leave-One-Out Validation (LOO): a cross-validation technique where each data point in the dataset is used once as the test set, while the remaining points are used for training. This can be useful when the dataset is small and cannot be split into a training and validation set efficiently. In some cases, combined approaches are possible and the strongest is probably the one where the dataset is split into training set and testing set, the training set is internally validated with cross validation, and the result is tested on the independent testing set. Last but not least, the major issue in medical statistics, the reproducibility of data and results, still holds in machine learning. Machine learning introduces an additional complication, as some techniques (e.g., RF, ANNs) are not fully deterministic and rely on stochastic seeds. For example, variable selection in random forest, aimed at perturbing the input data, is often stochastic. This means that two random forest models built on the same data can often be slightly different.

## 3. Machine Learning Applied to HSCT

Making a certain diagnosis of the patient’s illness is the first step in any clinical scenario, as it forms the basis for determining an accurate prognosis. Traditionally, clinical staff members conduct this phase, interpreting exams results and symptoms in accordance with the guidelines. In recent years, the progress in scientific research generated a huge amount of additional information useful for the clinical decision process. At the same time, all of this information is in some way confusing, and therefore it is difficult to give the correct relevance to each one of them. Here, the application of machine learning methods comes to the rescue, which allows the definition of the role of each component during clinical management. According to the literature, machine learning models have been tested in all phases of patient management. In the present review, we report and comment on what is published and emerging in PubMed, using “bone marrow transplantation machine learning” and “hematopoietic stem cell transplantation machine learning” as keywords. For the publication data, the results span from 2014 to 2024. The results of both studies were merged and duplicates were removed, leaving 82 articles for the analysis. Considering the main topics of the publications, in the present review, those are grouped into three categories: (i) decision tree support, (ii) mortality prediction, and (iii) post-HSCT complications.

**Figure 1:**
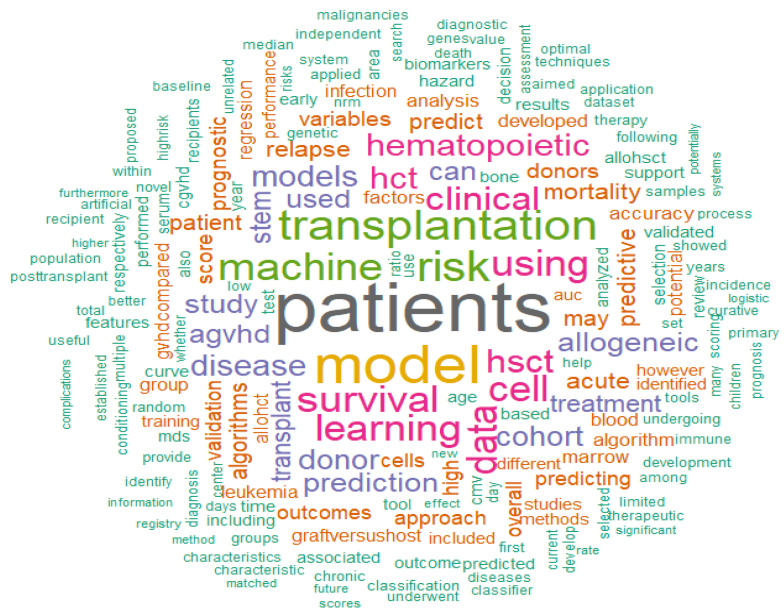
Word cloud reporting the main recurring words in the abstracts collected in the present review.

In terms of keywords, the collection of the abstracts uses the words illustrated in Figure 1.

**Figure 2:**
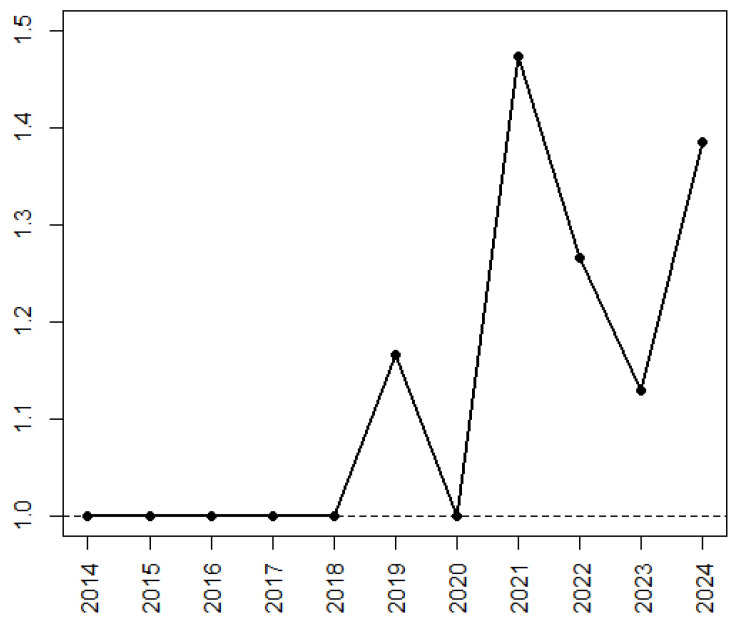
Scheme of the trend of the bibliometric indicators. On the X-axis, the years, and on the Y-axis, the average value of the quartiles of the sectors associated with the journals (https://www.scimagojr.com/ accessed on 31 December 2024).

In terms of bibliometric indicators, while the first papers were published in journals in the first Quartile (Q1), during the time the bibliometric indicators drop a bit, as indicated in Figure 2.

### 3.1. Decision Support

In this work, the concept of “decision support” has been intended as the whole group of choices that have to be made from the time of diagnosis to the last treatment given to the patient according to the scenario. Therefore, this section is divided into two additional subsections, one for each stage of the patient’s management: (i) diagnosis, and (ii) donor and conditioning regimen selection.

#### 3.1.1. Diagnosis

The diagnosis phase is unique to each condition, but for the most part, at least for hematological malignancies, it focuses on biochemical and morphological analysis of blood samples. Morphologic analysis does not always produce clear and precise results due to technical or biological reasons. For example, in cases of Acute Myeloid Leukemia (AML), if genetic abnormalities are not presents, the classification is based entirely on the morphologic aspects of the cell population [20]. As shown in the literature, machine learning methods allow for faster and more accurate detection of leukemic cells. In fact, Chandradevan et al. developed a model based on convolutional neuronal networks trained to detect AML and MM cells in bone marrow smears, with 87.2% and 96.5% of accuracy, respectively. This model combined a cell-detection algorithm (based on a Faster Region-Based Convolutional Network) capable of reading input images and detecting all cells using bounding box regression to predict bounding box locations, region pooling, and a residual convolutional network to extract a features map. A pre-trained model was used to initiate residual convolutional network, and 500 rounds of training refinement were performed. After the cells were identified, a new algorithm was created to categorize them into the correct cell type via a VGG16 convolutional network [21]. Another example of machine learning use is the diagnosis of Acute Promyelocytic Leukemia (APL), a kind of leukemia distinguished primarily by the presence of the t(15;17) *PML-RARA* translocation. Cytology is the quickest approach to diagnosing APL, followed by PML-RARA mRNA amplification for confirmation. However, in some circumstances, cytologic analysis is not an easy operation and mistakes might be made, especially when RARA is fused with proteins other than MPL (for example, NPM1 [22], BCOR [23], ZBTB16 [24]). In addition, APL is for the most part positive for the myeloperoxidase (MPO), but there are cases in which there is no expression of the protein [25], making it more difficult to correctly identify the disease. To address these challenges, Cheli et al. developed a machine learning-based open-source method for categorizing APL and distinguishing it from other AML subtypes. Their method integrated microscopic images of blood smears with some hematological characteristics, including fibrinogen levels, white blood cell count, prothrombin time ratio, mean corpuscular hemoglobin concentration, mean corpuscular volume, polynuclear neutrophil count, lymphocytes, and age. Researchers tested various models, including XGBoost, RF, gradient boosting classifier, Adaboost classifier, DT, LR, and SVM, but XGBoost’s gradient boosting algorithm performed the best in the test cohort, with an area under the receiver operator curve (ROC) of 0.95. The training cohort consisted of 177 patients, and the resulting trained model was tested in 4 retrospective groups for a total of 415 patients. The algorithm output was a combination of categorical (APL or non-APL) and numeric values, returning also the classification confidence score. This model allowed us to achieve 99.5% accuracy in 244 patients with a confidence score >99% and an accuracy of 85% in 114 patients with confidence score between 85 and 95% [26].

Machine learning has also been applied to predict the onset of Chronic Myeloid Leukemia (CML), therefore allowing for a diagnosis even before the symptoms manifest. Hauser et al. developed a predictive model in order to foresee the development of CML years before it happens, combining XGBoost and LASSO algorithms for variable selection, and tested two scenarios: one in which they used the model with the highest area under the curve (AUC) and another in which they tested the model with fewer variables selected. The data used in the work were extrapolated from the Veterans Health Administration database, which contains complete blood count data for the period between October 1999 and April 2020. Hauser and colleagues collected data 6 years prior CML diagnosis for a total of 1623 patients and analyzed those with the two models. The authors grouped data according to the time to CML diagnosis, identifying seven groups: 0, 6 months, 1, 2, 3, 4, 5 years to CML diagnosis. Their results revealed that using only basophil count as data, it is possible to help an increase in the AUC of models while reaching temporally close to the CML diagnosis, albeit the authors highlight the absence of model validation [27].

There have been additional cases where machine learning has been used for diagnostic purposes in onco-hematology. For example, ML has been tested on bone marrow samples from patients with myeloproliferative neoplasms (MPNs). In 2020, Sirinukunwattana et al. developed a machine learning model for automatically identifying and quantifying megakaryocytes in order to discover the specimens’ particular histological characteristics. The model was capable of achieving an AUC of 0.95 with the ability to distinguish between MPN subgroups [28]. The following year, Kimura et al. designed a model based on a convolutional neuronal network coupled with XGBoosting-based decision algorithm to combine histopathological images and complete blood counts of patients in order to identify cell subtypes and aberrant morphological features. Further training allowed us to distinguish between Polycythemia Vera (PV), Essential Thrombocytosis (ET), and Myelofibrosis (MF) MPNs subcategories with a sensitivity and specificity of >90% and AUC of ROC curves for PV, ET and MF of 0.990, 0.967 and 0.974, respectively [29].

#### 3.1.2. Donor Selection

Historically, hematopoietic stem cells were collected from bone marrow after pioneering works determined the presence of a hematopoietic stem cell niche in long bones [30,31,32]. After two decades of research, in the 1980s, the presence of hematopoietic stem cells outside the bone marrow, for example in the umbilical cord [33] and peripheral blood, was demonstrated.

Usually, the donor is first sought in the patient’s relatives because of the higher chances of being HLA matched. In cases of nonmatching, the search is expanded to unrelated matching donors through donor registry databases. This is a time-consuming process that, in some cases, requires up to 12 months, which can drastically reduce the survival rate [34]. Today, advances in databases and search algorithms have greatly improved efficiency, so much so that some searches can be completed in a matter of weeks. The donor search is skipped in case of auto-HSCT and therefore in this section the auto-HSCT will not be discussed. According to the EBMT survey [35], in Europe in 2021, 47,412 HSCT were performed, 42% of which were allogenic. In addition, the number of pediatric patients was 5437 (74% allogenic and 26% autologous). Globally, we have also reported increasing HSCT rates due to advances in multiple aspects, including the choice of donor [36].

Donor selection relies on specific immunological and clinical parameters. As previously anticipated, the main factor of patient–donor compatibility is the HLA matching. The human leukocyte antigen’s functions revolve around the induction and regulation of immune responses, together with the selection of a T cell repertoire. The HLA matching has an 8-point scale, in which 8/8 is the best possible matching and is given by the number of compatible HLA loci. The importance of HLA compatibility is due to the fact that matched donor cells should deliver competent proliferating cells able to cause graft versus leukemia (GVL), namely, a mechanism by which the donor’s immune cells target and eliminate the tumor cells. Unfortunately, there are cases in which the matching is not perfect (7/8 or less on the HLA matching scale) or there are differences in minor antigens that can lead to GVHD onset. Additionally, there are cases reported in which, even with a genetically identical donor (monozygotic twins), the risk of relapse is three times higher than transplantation from HLA-identical siblings [37]. Thus, the donor selection is a sort of compromise between 8/8 HLA-matching and having minor genetic differences with the patient, in a way that allows for graft versus leukemia without triggering GVHD (or triggering it but with low grades). The complexity of achieving optimal transplant outcomes is highlighted also by all these factors. In recent years, in part as a result of logistic limitations due to the COVID-19pandemic, the haploidentical-HSCT rate has strongly increased. The haploidentical HSCT is a particular allo-HSCT based on 50% of HLA compatibility, namely the match between first degree relatives [38,39].

Beyond HLA matching, when selecting a donor, another element to be considered is the donor’s age. According to studies, each decade of decrease in the donor age increases the overall survival (OS) by 3%, while other studies show that when treating elderly patients with HSCT, it is preferable to use a young matched unrelated donor over HLA-identical siblings [40,41]. Interestingly, even though the donor selection process relies on finding the youngest compatible subject, the patient’s 1-year post-HSCT OS seems to be unaffected by the donor’s characteristics [42].

Other donor selection criteria are sex matching, the donor serostatus for cytomegalovirus (CMV) matching and blood group. Sex matching should be preferred because male patients may develop GVHD due to the reactivity of female donor lymphocytes to antigens, especially if they have had pregnancies [43], encoded by the Y chromosome. CMV serostatus is important for CMV-negative patients because of the potential transmission and associated risks of receiving stem cells from a CMV seropositive donor; thus, a seropositive donor should be paired with a seropositive patient [44,45].

Given the complexity and heterogeneity of the factors involved in donor matching (genetic, clinical, and demographic data) and the impact that donor cells may have on post-transplant outcomes [46,47] many machine learning models have been developed to determine the best decision strategy. Many works can be found in the literature regarding this topic. Wadsworth et al., for example, developed a proportional odds model to create a useful and quick scoring system for predicting the likelihood of finding a matching donor based on HLA loci genotype frequencies [48].

Logan et al. implemented Bayesian machine learning algorithms in designing models for the optimal donor selection for HSCT. The authors trained the algorithm on a cohort of patients with AML, Acute Lymphoblastic Leukemia (ALL), CML, and MF transplanted in the United States between 1999 and 2014, with 8/8 HLA-matched unrelated donors, taking into account a long list of variables such as disease status, disease type, recipient age, gender, and CMV status, transplant variables, and donor clinical characteristics. The model was designed with a composite binary outcome of grade 3–4 GVHD or death by 180 days as negative outcomes. Model training was conducted on 10,318 patients and then validated on 3501 patients, and it returned an overall ranking of the variable’s selection probability as output. Donor age was identified as the variable with the highest relative selection probability among donor features, implying a higher likelihood of a positive HSCT outcome regardless of patient disease or transplant characteristics [49]. Similar work has been conducted by Buturovic et al. who created a multivariate Support Vector Machine (SVM) classifier with the intention to identify preferred unrelated donors for a given recipient with AML and ALL. The model included variables such as clinical patient data, transplant data, and clinical donor data, with the addition of donor telomere length. The outcome was set to be the survival to 5 years after HSCT. Despite the effort, the authors declared this work to be inconclusiveness, blaming a possible lack of variables that truly impact the HSCT transplant, and that a larger dataset could result in significant results [50].

As previously mentioned, there are different types of conditioning regimens, classified on the basis of the myeloablative power. In the literature, there are very few studies regarding the application of ML in the conditioning regimen decision process, and only one was found in our PubMed search. In this work, authors designed a regression model based on propensity score weight in order to identify the relevant features later selected with the LASSO regularization. This method allowed the formulation of a score (“benefit score for RIC”) defined as the sum of the interaction term between treatment (myeloablative (MAC) or reduced intensity (RIC) conditioning) and covariates. The analysis made it possible to identify a cut-off according to which to define patients suitable for RIC or MAC [51].

In addition to this, the potential of ML in this context also lies in its ability to analyze large datasets to make personalized decisions. For example, ML could integrate real-time clinical and genetic data to optimize regimen intensity while minimizing toxicity risks and maximizing the patient OS. In particular, ML models can implement the following: (i) HLA-matching algorithms to analyze large datasets in the attempt to provide most accurate and sensitive predictions for histocompatibility; (ii) non-HLA-related models, to investigate factors other than histocompatibility (e.g., age, sex, CMV serostatus, comorbidities, et cetera). The above-mentioned points are crucial in donor selection and, to date, represent an important challenge to improve the HSCT management and therefore ML, potentially, could both enhance and facilitate the donor selection process.

### 3.2. Mortality/Relapse Prediction

The tumor reduction or cleanse is one of the most important outcomes of HSCT, but not the only one. In fact, in addition to the responsiveness of the transplant, there are other parameters that are accounted for when evaluating the success of the HSCT. When dealing with bone marrow transplantation (and other treatments), it is important to evaluate the engraftment, the GVL effect and the probabilities of OS, treatment-related mortality (TRM), non-relapse mortality (NRM), disease-free survival (DFS), and relapse-free survival (RFS). Our research returned articles with predominantly overall survival and relapse risk as main themes. In fact, many studies revolve around the development of models for risk prediction and stratification that take into account a variety of variables, including clinical, genomic, and demographic data.

In 2014, Armand et al. published their work on the definition of a disease risk score for allo-HSCT. Combining original guideline stratification, clinical, and transplant features via a multivariate regression model, they were able to design a risk score on which to stratify patients according to the new score for cases with ALL, AML, CLL, CML, MDS, MM, MPNs, and various types of lymphomas. They identified four categories: low, intermediate, high, and very high risk, with the respective OS until 24 months after HSCT. The authors discussed the need to implement a bigger dataset comprehensive of more types of data, for example, genomic data [52].

The following year, Shouval et al. published their data mining work regarding allo-HSCT 100-day mortality prediction score in AML patients. They designed a model based on logistic transformation functions, using data from 28,236 patients recorded in the AL registry of the Europe Group of Blood and Marrow Transplantation. Their analysis allowed them to create an alternating decision tree based on the clinical data of patients and donors, giving a continuous prediction score for each of the tree branching points [12].

Two years later, they refined their prediction score by introducing ranges of values: low, intermediate, and high level. Consequently, they analyzed the impact of the three categories on OS, leukemia-free survival (LFS), and NRM at 1- and 2-years post-HSCT, using the low-risk category as a reference value. Following the ML algorithm classification, the hazard ratios of the above parameters are directly associated with the prediction score; this means that higher the prediction score, higher the hazard ratio [53].

Similar work has been carried out by Fuse et al., who applied ADTree to make an accurate after allo-HSCT relapse prediction [54].

Shouval et al. also published a technical article in which they compared six different ML models (AdaBoost, ADT, LR, ANN, NB, and RF) in order to assess which, one is the best data fitting in predicting the mortality at 100 days after HSCT. Including the most common patient, donor, and transplant variables as inputs, the best fitting model resulted in the Adaboost with an AUC of 0.67, together with the random forest model. Interestingly, basically all models returned similar results, with only hundreds of differences. According to the authors, it is possible that using “traditional” clinical HSCT data can get to a point of predictive saturation, making it virtually impossible to design better models with the same inputs. Therefore, they suggest an enlargement of data, covering also genetic, clonal, and biological factors [55].

The following year, Marino et al. reported their results on what Shouval and colleagues suggested: a multivariate logistic regression model of OS, DFS, and TRM including genetic data. Their model used specific aminoacidic substitutions in HLA genes and other variables (age, sex, disease, transplant, and conditioning) as inputs, classifying amino acid substitutions into high risk and non-high risk, giving an adjusted estimate for the OS, DFS, and TRM [56].

Genomic data were also taken into account by Ritari et al. during the development of a random forest classification model in order to define the impact of genetic variants on the relapse risk. Patient germline genetic features were evaluated via whole-exome sequencing and were initially analyzed together with donor age, diagnosis, and graft type as covariates. From this analysis, only variants with a *p*-value < 0.001 were selected as inputs for the RF classification model. Their model showed an AUC of 0.717 in the training cohort and a mean AUC of 0.670 in the two replication datasets. In both replication cases and also in the training set, the *p*-values associated with the ability to correctly predict relapse were <0.001 [57].

The published study shows genomic and clinical data being analyzed in order to predict OS in patients with MDS using a fit model that outperformed the standard International Prognostic Scoring System (IPSS) and returned the OS probability for each of the mutated genes at diagnosis [58]. Orgueira et al. evaluated the performance of multiple models according to the number of variables included and compared the results with the standard IPSS. ML models showed better fitting throughout the whole timeline when evaluating OS prediction [59].

Goswami et al. applied a stacked ML model to predict relapse within 3 years of autologous bone marrow transplant in patients with MM. They used spectral clustering to divide patients into low- and high-risk groups (relapse within 36 months was considered high risk, otherwise low risk), and then calibrated the relapse risk groups [60].

By combining self-organizing maps, t-distributed stochastic neighbor embedding, and marker enrichment modelling, Gandelman et al. developed a risk stratification algorithm for transplant patients that took into account their comorbidities affecting the gastrointestinal tract, liver, eyes, joints, and lungs. This allowed for the identification of three risk categories (low, intermediate, and high), which were based on the survival probability [61].

AML patients’ specific blast features can be identified by Arabyarmohammadi et al.’s machine learning model, which enables relapse prediction. They based their work on a u-net deep learning model to identify the blast cells and a LASSO algorithm to extract all the features. The analysis was performed on specimens of bone marrow after HSCT and showed different textural appearance of myeloblasts of patients who relapsed compared to those who did not [62].

Lee’s group took into account clinical, transplantation, and genomic data in AML patients, elaborating them with four different models: SVM, LR, Bidirectional Long Short-Term Memory (BLSTM), and Attention-based BLSTM (Att-BLSTM, designed by the authors). The aim was to predict mortality and relapse probability. Between the four models, Att-BLSTM achieved the highest AUC for both mortality and relapse probability, 0.77 and 0.67, respectively [63].

ML has also been applied in the interpretation of Positron Emission Tomography (PET) of patients with Hodgkin lymphomas in order to predict if the patient has low or high risk of disease progression, allowing for a prompt change in therapy in cases where it is needed [64].

Numerous studies have been conducted on the design and application of ML algorithms, including pathologies as categorical variables, and therefore the results are not directly comparable. Iwasaki et al. showed a low contribution of diagnosis and disease stage at HSCT to RFS probability, whereas the disease risk index (DRI) and disease stage are the most impactful ones on the OS [65].

Other works did not consider the disease at all, but instead they studied the impact of HSCT, regardless of the pathology. For example, McCurdy et al. reported their results on the application of a classification and regression tree for OS treated as a time-to-event outcome. In fact, the authors evaluated the impact of natural killer cells (NKCs) count on the OS, PFS, relapse, and NRM probability, identifying a cut-off of 50.5 cells/uL as the one capable of distinguishing 2 different trends in each of the 4 parameters. To be more specific, having a NKCs count greater than the above cut-off increases the probability of OS and PFS while reducing the probability of relapse and NRM. The additional random forest model confirmed NKCs count as the most important variable affecting the OS probability [66]. As mentioned, ML represents an innovative and promising tool in predicting outcomes in stem cell transplantation, in particular (i) relapse and (ii) mortality prediction. In the first case, ML can be used to identify patterns in pre- and post-transplant data that correlate with relapse risk, while in the latter, it can predict OS based on algorithms like Cox proportional hazards models. Therefore, a correctly structured model could provide a useful predictive power to optimize patients’ management, especially in enabling earlier interventions.

### 3.3. Post-HSCT Complications

HSCT can have various types of complications, some of which are related to the conditioning regimen toxicities, such as mucositis, lung disorders, or sinusoidal obstructive syndrome, and some of which are related to the myeloablative effect of the conditioning drugs, which lead to a much higher probability of developing fungal, bacterial, or viral infections. Among those there is graft failure, which can lead to prolonged neutropenia, thrombocytopenia, and anemia, which increase the risk of bleeding, infection, and other severe complications, and acute/chronic GVHD can also occur.

The majority of the articles included in our research referring to HSCT complications revolve around the occurrence of acute or chronic GVHD, since this is not a rare event post-HSCT. In fact, 30–60% of transplanted patients develop aGVHD and 30–70% develop cGVHD.

Many works described the performance differences of various ML algorithms in defining the likelihood of aGVHD development. Cirruse Salehnasab et al. demonstrated that eXtreme Gradient Boosting Classifier (XGBClassifier) has a greater AUC compared to HistGradientBoosting, ADABoost and random forest classifiers when predicting the impact of hematological parameters on the aGVHD onset (90.98 vs. 90.65, 85.33, 83.48) [67]. Whereas, other approaches, such as linear discriminant analysis (LDA), KNN and multilayer perceptron (MLP), have been implemented to predict the grade of aGVHD according to the number of features included [68]. Similar work has been conducted by a Japanese group using five different models: NB, ADTree, MLP, RF and Adaboost. Of these five models, ADTree performed as the best predictor for grade I-II aGVHD (AUC = 0.616) and scored the same AUC of NB in predicting grade III-IV aGVHD (AUC = 0.622) as the best predictors for this scenario. Moreover, their approach allowed for the production of two complete decision trees: one for grade I-II and one for grade III-IV aGVHD [69]. Moreover, logistic regressors and convolutional neural networks can also be used in the design of prediction models on the same theme [69,70]. All the above works used patient and transplant clinical data, but there are other features that have been included in other analyses, for example, the RNA expression levels of specific genes via a NB approach, as shown by Rowley et al. They performed RNA expression analysis of 1402 genes pre- and post-HSCT on bone marrow samples and compared the results. Of all the 1402 genes analyzed, only 92 showed a correlation with aGVHD onset, with an AUC of 0.721 when using post-HSCT data, the ones found to be more predictive [71]. Furthermore, it has been proven that ML can also predict the impact of post-HSCT antibiotic therapy on the development of aGVHD [72]. In the same way, various ML algorithms are used to predict cGVHD risk [73,74,75,76].

The risk of developing other HSCT complications, such as infections, kidney injuries, hepatic venous–occlusive disorder, venous thromboembolism, or even lymphoproliferative pathologies, has been assessed in various models [77,78,79,80,81,82].

Infections are the most common HSCT complications due to the myeloablative conditioning regimen [83] and this can lead to infections from external pathogens or from the activation of common viruses such as CMV [84] or Epstein–Barr [85], or bacterial sepsis [86]. Another example is the work of Montassier et al., who sequenced the 16S ribosomal RNA of non-Hodgkin lymphoma patients gut microbiome in order to quantify bacterial taxa and later identify, via ML model, highly impacting biomarkers related to bloodstream infections (BSI). They implemented a 500-tree RF model in order to predict the likelihood of developing BSI based on the gut microbiome taxa [87]. Generally, the bacterial infection risk is managed with antimicrobial prophylaxis and treatment if infection actually develops. However, in a recent study by Rashidi et al., antimicrobial treatment has been demonstrated to promote the development of aGVHD within 30 days from transplant. The type of antimicrobial drug can lead to different time and risk of aGVHD onset [72].

As described in the above paragraph, both acute and chronic GVHD represent an important complication that can reduce transplant success probability. ML models can help clinicians to stratify patients based on their risk of developing GVHD and therefore define an optimum GVHD prophylaxis and, eventually, the best available therapy based on patients’ characteristics.

### 3.4. Evolution of ML Application and Techniques During the Time Period

**Figure 3:**
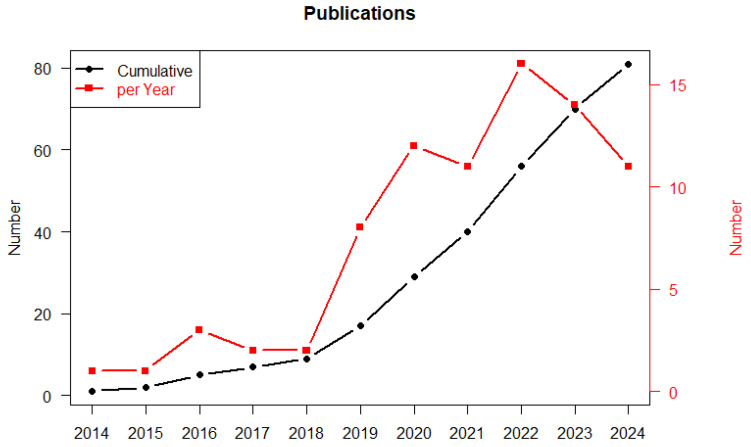
The graph reports the number of absolute and cumulative publications over time in the last 10 years (2014–2024).

The figure below (Figure 3) shows the publications during the time, absolute and cumulative. Even if with some fluctuations, the application of ML to HSCT is clearly growing.

**Figure 4:**
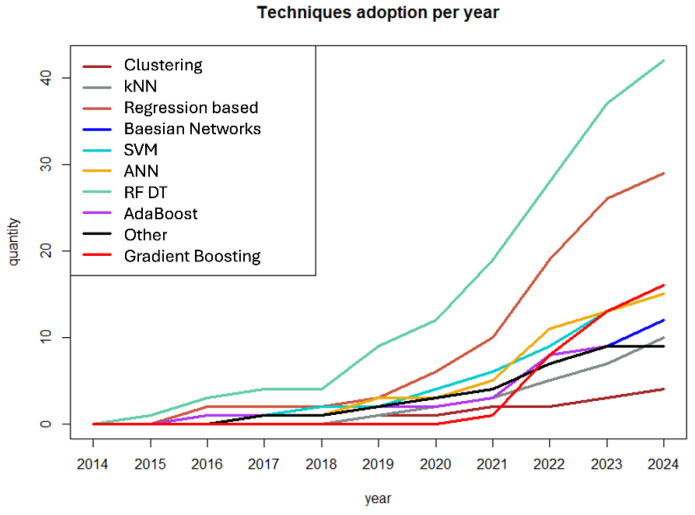
Distribution of the application of different ML algorithm types to HSCT per year. The time frame considered was 2014–2024. kNN = k-Nearest Neighbors, VM = Support Vector Machine, ANN = Artificial Neural Network, RF-DT = random forest (RF) and decision trees (DT).

About the ML techniques, DT/RF are the most adopted techniques, followed by the regression-based techniques. Surprisingly, ANN are still relatively unexplored, probably due to the high quantity of data they require to be trained successfully (Figure 4).

**Figure 5:**
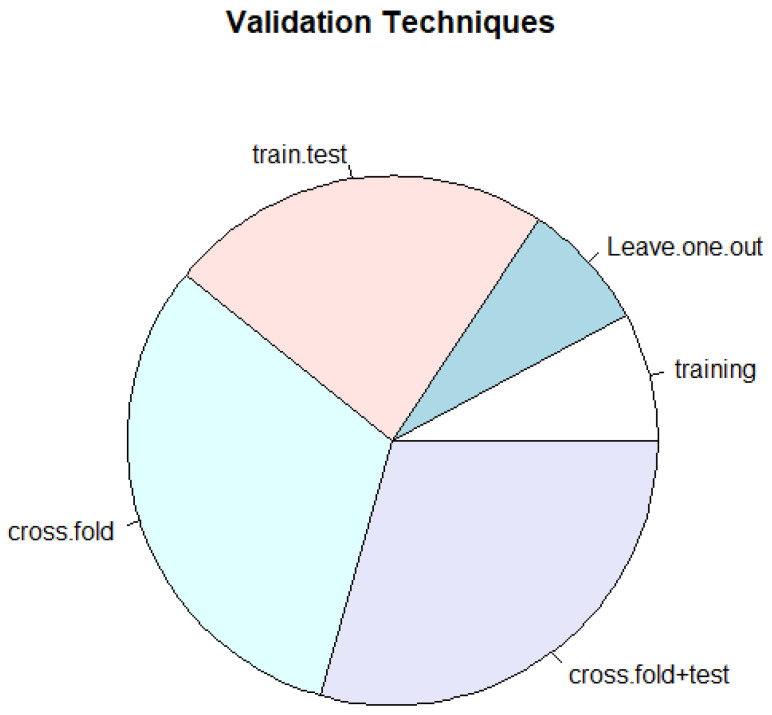
Types of ML validation techniques applied in publications included and commented on in the present review.

Regarding the validation, the quality seems globally reasonable: only in a few cases was the training test also used for validation. In more than fifty percent of the cases, at least a train/test or a cross-fold validation was used (Figure 5).

## 4. Challenges and Limitations

Despite a lot of studies and attempts being made, there is much more to do in order to better define and regulate ML applications. The analysis of the articles included in this work allowed us to collect challenges and limitations commonly described by various authors. Challenges have been found in the data collection phase, in which numerous data types are included in the clinical research, together with the lack of a standardized file format, the collection system and inconsistencies in the labelling and annotation of the data make collecting ready-to-use data impossible, also considering the high number of missing records. Therefore, data completeness and quality have to be sought, although this usually leads to the creation of huge and high-dimensional datasets of difficult interpretations, often without considering the presence of confounders. In addition, the integration of multi-modal data, such as the combination of clinical, genomic, and imaging data, poses further technical and computational challenges. Algorithm development is no easy task, and it is possible to make errors during the design phase. As for canonical clinical studies, if the study and data collection concept are not well-designed, biases can manifest, and the same can happen for ML algorithm development, together with the overfitting. With that being said, comparing ML application results found in the literature is difficult and sometimes useless due to the fact that different study groups use different data types and different algorithms. In addition, sometimes it is hard to interpret ML results, leading to a lack of application in clinical decision support. In addition, the reproducibility and comparability of results between studies is limited by the lack of established benchmarking standards. Finally, the widespread use of ML algorithms in a variety of fields appears to jeopardize ethical and social concerns centered on the protection of individual privacy rights by encouraging the free exchange of personal data.

ML models are often considered superior to statistical models, but we believe that this statement is difficult to verify for several reasons. For instance, the concept of validation is often radically different: in statistics, the sample size itself is a primary element that provides confidence in the statistical model, allowing the calculation of statistical power. Conversely, ML tends to validate models a posteriori (often without estimating the sample size), which raises important considerations when comparing results.

Moreover, performance indicators can differ (e.g., *p*-value vs. mean GINI index) and are not directly comparable.

Last but not least, model simplicity and interpretability are often important qualitative factors, which, in the case of some ML models (e.g., ANNs), are sacrificed in favour of seemingly better performance indicators.

In essence, the authors’ opinion is that, yes, ML techniques appear more performant, but it will still take time for (a) this superiority to become more clearly evident and (b) users to fully understand and correctly interpret the results provided by ML models.

These challenges enlighten the limitations that block, or at least slow down, the development and application of ML algorithms. The lack of interpretable predictive models and multi-center studies is leading to biased and non-generalizable models on small sample size cohorts, more often when the validation step is not included. Machine learning models, applied to HSCT but not limited to, can benefit from multi-center studies given the larger data collected and analyzed compared to a single center experience, allowing a better trained and validated model. In addition, laws and regulatory rules are still lacking in some fields, giving space to privacy concerns to develop. A concerted effort by researchers, clinicians and other healthcare personnel to establish a robust framework for the implementation of ML in healthcare is needed to address these limitations.

## 5. Not Only Models: Infrastructures, Legal Issues, and Data Harmonization

Looking to the future, the potential of AI implicitly demands addressing other satellite but crucial issues. One of these is certainly the development of software infrastructures to support research, enabling the paradigms of rapid learning [88] and federated learning [89]. The former allows the training of models that are automatically updated with new evidence, while the latter facilitates the implementation of multicenter studies capable of training and validating models across independent testing sets without requiring data to leave the hospitals where it was collected.

This latter point, in particular, aligns with the increasing emphasis that legislation places on protecting patient privacy and safeguarding sensitive data. At the current state of the art, this concern is best addressed through a federated learning approach.

Some technological infrastructure proposals, both for federated learning and rapid learning, have been introduced, but they are relatively new, and a solid standard has yet to be established [90,91,92,93].

**Figure 6:**
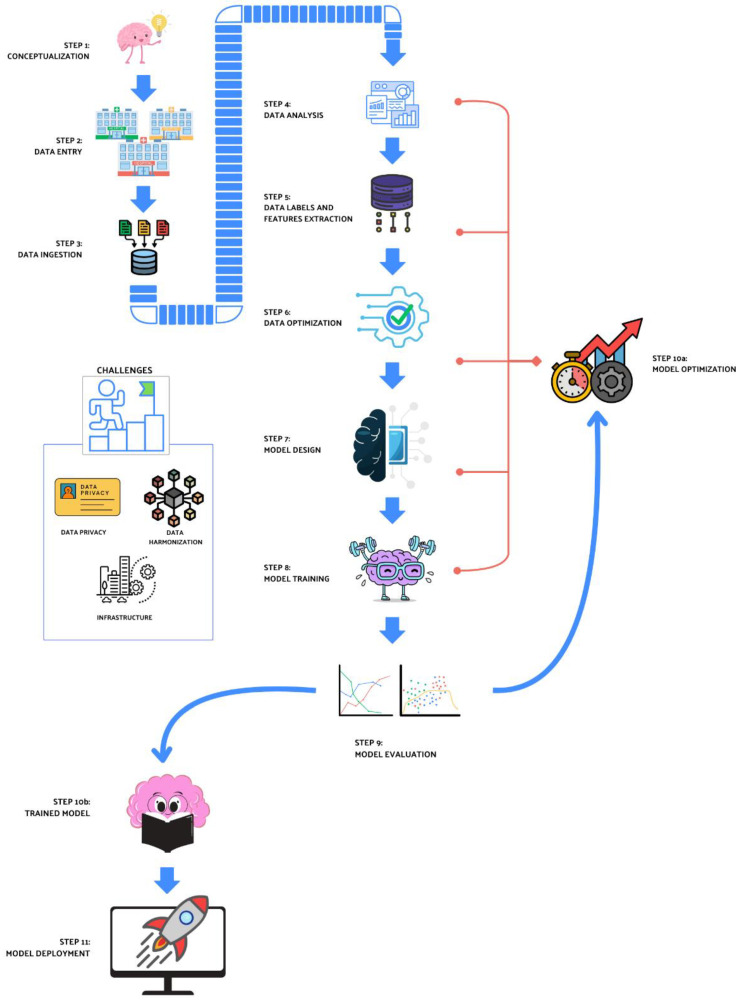
Example of machine learning workflow. The figure above shows the multiple steps involved in general clinical ML applications. Step 1 to step 11 are as follows: (1) initial conceptualization of scientific rationale; (2) data entry step, which can occur from single to multiple centers; (3) data ingestion step, that feeds data from multiple sources; (4) data analysis, which consists in the first data cleaning step evaluating data compliance; (5) data labels and features extraction, for the identification of output categories and variables; (6) data optimization, an additional step to fit data with scientific rationale, label and features; (7) model design, in order to create the desired ML model; (8) model training, feeding data to the designed models; (9) model evaluation, quantify accuracy and sensitivity of the designed model to assess model behavior. If the statistical power and the significance are not confirmed, it is possible to undergo the (10a) model optimization step. At this point, the optimization phase can start from any of the steps from 4 to 8, in order to satisfy the statistical requirements; (10b) the trained model, achieved when statistical analysis are successfully completed; (11) model deployment, when the model is fit to the scientific rationale, the available infrastructure and current legislation. Lastly, current challenges are reported in the corresponding box (in the middle-left): (i) legal aspect with particular emphasis on data privacy rights protection; (ii) data harmonization and (iii) infrastructure.

Such infrastructures, potentially capable of providing ML models with a vast pool of data for training, still need to expose harmonized data in terms of type and format, mapped onto standard ontologies. In this regard, specific consortia (e.g., openEHR, CDISC, OHDSI/OMOP) are working to build standard models for the formal representation of aspects of the world (e.g., clinical trials, generalist or disease-related data models). However, much work remains to be performed, especially for newer biomarkers or relatively recent omics analyses (e.g., radiomics). A diagram concerning the ML workflow together with the main challenges is reported in Figure 6.

## 6. Conclusions and Future Perspectives

This work aimed to describe the development status of ML techniques in the HSCT setting, giving insight into what has already been conducted in various onco-hematological diseases. The majority of the studies analyzed here used DT/RF algorithms and focused on the application of ML to the definition of a risk and prognostic score. There is a dearth of literature on the development of decision support trees for conditioning regimens, so more research is necessary to pinpoint influential characteristics, also because, as already demonstrated, conditioning intensity can impact transplant outcome [94,95]. Therefore, extensive work is necessary in this context, also looking at new parameters that are expected to impact HSCT outcomes, such as psychological aspects, nutritional status and the quality of life of patients and care givers [96,97,98]. We see the need for starting projects with a larger sample size and including different types and more numerous features, such as clinical, demographic, genomic, and molecular. In this way, it would be possible to have a better view of features contribution, in each specific disease, to diagnosis, risk, and outcome prediction, always keeping an eye on possible confounders that may lead to errors. As shown in the literature, many factors can contribute to disease assessment and outcome, and different types of algorithms run differently, giving no chance to define the best overall model without large multicentric independent validation. In addition, the lack of standardized methodologies and the diversity of algorithms used in the studies make it difficult to make meaningful comparisons or to reach a consensus on the most effective approaches.

## Figures and Tables

**Table 1:** Different categories of algorithms used in machine learning.

Technique	Description
k-Nearest Neighbors (kNN)	The k-Nearest Neighbors (kNN) algorithm is a simple, non-parametric machine learning method used for classification and regression. It predicts the output for a given input based on the majority class or average of its closest k training samples in the feature space.
Linear regression (LR)-based techniques	They are a family of ML learning techniques where a linear regressor is trained and can be used, by a threshold, as a classifier. In this family we have the linear regression, logistic regression, LASSO, etc.
Support Vector Machine (SVM)	They are supervised machine learning algorithms used for classification and regression. They work by finding the optimal hyperplane that maximizes the margin between data points of different classes, often using kernel functions to handle non-linear separations.
Bayesian Network (BN)	Bayesian networks are probabilistic graphical models that represent relationships among variables using nodes and directed edges. They encode conditional dependencies and allow for reasoning under uncertainty by applying Bayes’ theorem to update beliefs based on new evidence.
Decision Trees (DTs) and Random Forest (RF)	DT is an algorithm used for classification and regression that splits data into branches based on feature values. It works by recursively partitioning the input space to create a tree structure, where each node represents a decision and each leaf a final prediction. RF is an ensemble learning approach exploiting many DTs (often more than 500), differently trained, to estimate the most probable result.
Artificial Neural Network (ANN)	An Artificial Neural Network (ANN) is a machine learning model inspired by the structure of the human brain, consisting of interconnected layers of nodes (neurons). It processes input data through weighted connections and activation functions to learn complex patterns for tasks like classification, regression, and more.
Gradient Boosting and Adaboost	They are ensemble learning algorithms that combine multiple weak classifiers, often decision trees, to create a strong classifier. Each new model corrects the errors of the previous ones by optimizing a loss function, resulting in a strong predictive model. During the learning, Adaboost emphasizes reweighting samples, while Gradient Boost emphasizes reducing residual errors through gradient optimization.
Clustering	Clustering is an unsupervised machine learning technique that groups similar data points into clusters based on their features. The goal is to identify inherent structures in the data without predefined labels, enabling pattern recognition and data segmentation.

## Data Availability

Data are available from the corresponding authors upon request.
